# Functions of Presynaptic Voltage-gated Calcium Channels

**DOI:** 10.1093/function/zqaa027

**Published:** 2020-10-23

**Authors:** Annette C Dolphin

**Affiliations:** Department of Neuroscience, Physiology and Pharmacology, University College London, WC1E 6BT, UK

**Keywords:** calcium channel, biophysical properties, molecular properties, auxiliary subunit, presynaptic terminal, synapse, voltage-gated, second messenger

## Abstract

Voltage-gated calcium channels are the principal conduits for depolarization-mediated Ca^2+^ entry into excitable cells. In this review, the biophysical properties of the relevant members of this family of channels, those that are present in presynaptic terminals, will be discussed in relation to their function in mediating neurotransmitter release. Voltage-gated calcium channels have properties that ensure they are specialized for particular roles, for example, differences in their activation voltage threshold, their various kinetic properties, and their voltage-dependence of inactivation. All these attributes play into the ability of the various voltage-gated calcium channels to participate in different patterns of presynaptic vesicular release. These include synaptic transmission resulting from single action potentials, and longer-term changes mediated by bursts or trains of action potentials, as well as release resulting from graded changes in membrane potential in specialized sensory synapses.

## Introduction

Voltage-gated calcium (Ca_V_) channels are well understood to function as the route for Ca^2+^ entry into cells, particularly excitable cells, in response to depolarization. However, they represent a family of channels with a variety of biophysical properties that are exploited differentially to perform particular functions in presynaptic terminals. These varied roles will be explored in relation to different types of synaptic boutons. It is important to understand how the membrane potential of the presynaptic terminal, which is dictated in part by other channels present, as well as the intracellular free Ca^2+^, affects the dynamics of the Ca_V_ channel activity. Their properties, in addition to the positional anchoring of the particular channels, dictate their ability to trigger and sustain vesicular release.

## Molecular properties of Ca_V_ channels

Distinct voltage-dependent Ca^2+^ conductances were first characterized by electrophysiological and pharmacological means, involving both whole-cell and single-channel recording. A number of different currents were identified,^1–3^ and termed L-type,^4^ T-type, or low voltage-activated,^2^^,^^4^ N-type,^4^ P-type,^5^ and R-type^6^ (Table 1). Subsequent molecular cloning identified three sub-families of mammalian Ca_V_ channels: Ca_V_1 with four members (all of them giving rise to L-type currents), Ca_V_2 with three members (forming P/Q-, N-, and R-type currents), and Ca_V_3 with three members, all producing T-type currents (Table 1).

**Table 1.** Properties and Voltage-Dependent Activation of Ca_V_ Channels

	Gene	Name When Cloned	Systematic Protein Name	Physiological Name	V_50_, _activation_ Using 1–4 mM Divalent Cation (except Ca_V_1.4: 15–20 mM)	Physiological Function	Function in Synaptic Transmission
HVA	*CACNA1S*	α_1_S	Ca_V_1.1	L		Mechanical coupling with SR; skeletal muscle contraction	None known
	*CACNA1C*	α_1_C	Ca_V_1.2		−18 mV (mouse) in 2 mM Ca^2+^.^7^	Cardiac/smooth muscle contraction; hormone secretion	Long term processes e.g. LTP in hippocampal mossy fibers^8^
	*CACNA1D*	α_1_D	Ca_V_1.3		−39 mV (rat) in 2mM Ca^2+^.^7^ −9.4 mV (human) in 2 mM Ca^2+^.^9^	Secretion of hormones, sinoatrial node function	Auditory hair cell synaptic transmission
	*CACNA1F*	α_1_F	Ca_V_1.4		−4 mV (human, full-length) and −18 mV (Δ exon 47) in 20 mM Ba^2+^.^10^ +0.6 mV (human) in 15 mM Ca^2+^.^11^	Retinal transmission in photoreceptors and bipolar neurons	
	*CACNA1A*	α_1_A	Ca_V_2.1	P/Q	−5.7 mV (rat) in 1 mM Ba^2+^.^12^ +4.9 mV (zebrafish) in 2 mM Ca^2+^.^13^	Neuronal, mainly presynaptic	
	*CACNA1B*	α_1_B	Ca_V_2.2	N	−5.7 mV (rabbit) in 1 mM Ba^2+^.^12^ −1.3 mV (zebrafish) in 2 mM Ca^2+^.^13^ −13 mV (rat) in 2mM Ca^2+^.^7^	Neuronal, mainly presynaptic	
	*CACNA1E*	α_1_E	Ca_V_2.3	R	−29 mV (rat) in 4 mM Ba^2+^.^14^	Involved presynaptically, particularly in asynchronous release	
LVA	*CACNA1G*	α_1_G	Ca_V_3.1	T	−47 mV (rat) in 2mM Ca^2+^.^7^ −45.5 mV (rat) in 1.25 mM Ca^2+^.^15^	Subthreshold and oscillatory behavior in neurons and other excitable cells	
	*CACNA1H*	α_1_H	Ca_V_3.2		−45.8 mV (human) in 1.25 mM Ca^2+^.^15^		Present in some synapses
	*CACNA1I*	α_1_I	Ca_V_3.3		−43.8 mV (rat) in 1.25 mM Ca^2+^.^15^		

The table describes the 10 mammalian Ca_V_ genes products and collects data on V_50, activation_ from a number of original sources. It is necessary to refer to the papers for details of the auxiliary subunits, splice variants, and other conditions used, which can further affect the biophysical properties of the channels. LVA and HVA refer to the original nomenclature defining two types of calcium current (low- and high-voltage-activated).^2^

The pore-forming Ca_V_ α_1_ subunits all have very similar structures with 24 transmembrane segments separated into four domains, each with a voltage-sensing and a pore module.^16^^,^^17^ The domains are joined by intracellular loops, and a long C-terminal tail. The Ca_V_1 and Ca_V_2 channel α_1_ subunits are each associated with an auxiliary ß and α_2_δ subunit. There are four ß and four α_2_δ subunits, which have divergent cellular expression patterns, and confer some differing properties on the channels with which they associate (see below). The Ca_V_2 channels, particularly Ca_V_2.1 and Ca_V_2.2 are the main channels involved in presynaptic function.

## How the Biophysical Properties of Ca_V_ Channels Can Shape Their Function

**Figure 1.**
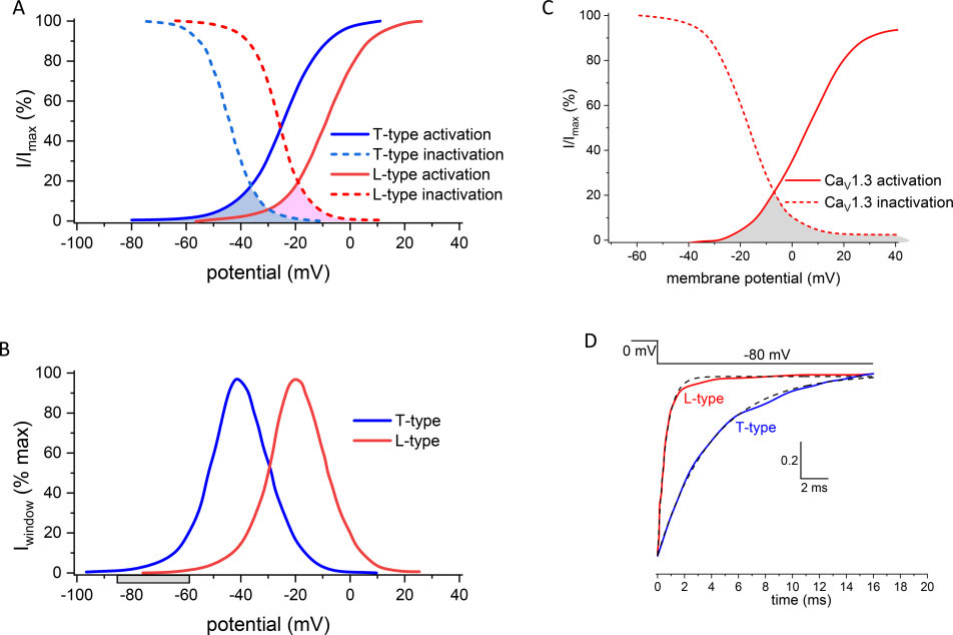
Idealized Voltage-Dependence of Activation and Inactivation for Selected Ca_V_ Channels. **(A, B)** Voltage-dependence of normalized activation (solid line) and inactivation (dotted line) of approximated T (Ca_V_3.1/2, blue) and L-type (Ca_V_1.2, red) currents, with window currents shaded in A, and replotted in B. Gray bar in B shows range of resting membrane potentials. Adapted from Fig 1b in Rossier.^18^**(C)** Data for Ca_V_1.3 digitized and replotted from Fig 5a,^9^ in which 15 mM Ca^2+^ was used as charge carrier, which shifts activation about +14 mV, compared to 2 mM Ca^2+^ (see Supplementary Table 3 in Azizan et al.^9^). **(D)** Normalized tail current data digitized and replotted from Fig. 2d in Carbone and Lux,^19^ showing the relative inactivation rate of L-type and T-type Ca^2+^ currents recorded from embryonic chick sensory neurons on repolarization to −80 mV in 5 mM Ca^2+^. The time constants of the tail currents, fitted by single exponentials (dotted lines) were ∼4 ms (T-type) and ∼0.6 ms (L-type).

Ca_V_ channels have a variety of characteristics that will be considered in this review, including voltage-dependent, kinetic and Ca^2+^-dependent properties (Table 1, Figure 1). The complex interplay between these elements determines the amount and timing of Ca^2+^ entry that occurs during depolarization, for example, during an action potential.

Voltage-dependent activation dictates the range of voltages over which the channels will activate when depolarized, which, for presynaptic terminals, is key to their excitability. Although Ca_V_ channels were originally divided into low (Ca_V_3) and high (Ca_V_1 and Ca_V_2) voltage-activated channels, it is clear that there is actually a continuum of activation ranges between these channels, when they are compared under more physiological conditions (Table 1). Such comparisons are nevertheless difficult to equate with physiological activation of these channels in neurons, and more specifically in presynaptic terminals. This is in part because the specific mix of channel splice isoforms^10^^,^^20^ and the associated auxiliary subunits associated with each channel, which can strongly affect their biophysical properties (Table 1), are rarely known. It is also the case that studies of the biophysical properties of Ca_V_ channels necessarily use non-physiological conditions to isolate the calcium currents, together with a variety of divalent cation concentrations (Table 1), which affect membrane charge screening to differing extents, and therefore influence the voltage drop across the membrane experienced by the channels.

Since voltage-dependent inactivation also occurs for most Ca_V_ channels over a range of physiological voltages, which are for the most part more negative than their activation range, the resting potential will determine the proportion of channels available to open. This availability will be different for each channel type; furthermore, in the resting membrane potential range of most neurons, or during small subthreshold depolarizations, only T-type currents will have the ability to exhibit any significant Ca^2+^ entry, termed the window current (Figure 1A, B).

Some Ca_V_ channels exhibit full voltage-dependent inactivation (Figure 1A, B), whereas for others it is incomplete (Figure 1C), meaning that a small proportion of the channels remain available for extended periods at depolarized potentials. This is particularly relevant to the functioning of the slowly inactivating L-type channels, Ca_V_1.3 and Ca_V_1.4, in specific presynaptic terminals in the inner ear and retina, respectively (see below). In addition to voltage-dependent inactivation, a second Ca^2+^-dependent inactivation process is important for some channels, and this may be triggered by global Ca^2+^ levels or local Ca^2+^ entry.^21^ The activation and inactivation of particular channels, as well as other properties, can be influenced by differential splicing,^10^^,^^22–24^ by auxiliary subunit composition,^22^^,^^25^^,^^26^ and by Ca^2+^-binding protein interaction.^21^^,^^27–29^ Although the inactivation processes may be too slow to affect Ca^2+^ entry during most presynaptic single action potentials, they can strongly influence Ca^2+^ entry over the course of action potential trains or bursts, and at specialized retinal and auditory synapses in which continuous Ca^2+^ entry occurs, which is modulated in a graded manner by membrane potential (see, for example, Ohn et al.^30^).

An important point that is infrequently considered is the deactivation rate of channels in response to repolarization of the membrane potential, since, together with activation rate, this can dictate the amount of Ca^2+^ entering a presynaptic terminal, as the extent of Ca^2+^ entry, particularly during a brief action potential, will be strongly affected by the rate of Ca_V_ channel closing. T-type channels have a slower deactivation rate, which is also voltage-dependent, being longer at more depolarized potentials,^19^ whereas for Ca_V_1 and Ca_V_2 channels, the deactivation rate is much more rapid (Figure 1D). Another key feature is the driving force for Ca^2+^ entry, dictated both by the Ca^2+^ concentration gradient and the membrane potential of the terminal.

Skeletal muscle calcium channels (Ca_V_1.1 or α_1_S) are unusual in that they act primarily as voltage sensors via mechanical coupling to open ryanodine receptors on the sarcoplasmic reticulum, a direct process not involving Ca^2+^ entry.^31^^,^^32^ Activation of the Ca_V_1.1 ionic conductance is very slow, relative to movement of its voltage sensors,^32^ and therefore Ca^2+^ entry is negligible during a single action potential. However, there is no clear evidence for significant functional expression of Ca_V_1.1 in neuronal tissue or for any presynaptic function.

## Multiple Roles of Ca_V_ Auxiliary Subunits

The ß and α_2_δ auxiliary subunits of calcium channels increase the transport of Ca_V_ channels to the plasma membrane, and this is particularly relevant to ß subunits, which prevent endoplasmic reticulum-associated proteasomal degradation of the Ca_V_ α_1_ subunits.^33^^,^^34^ Subsequently, there is an additional trafficking effect of α_2_δ subunits.^35^ The auxiliary subunits also confer a variety of properties on Ca_V_1 and Ca_V_2 channels; for example, certain splice variants of ß2 (ß2a and ß2e) slow the inactivation of Ca_V_1 and Ca_V_2 channels and are themselves membrane-associated.^36–38^ The α_2_δ subunits generally increase Ca_V_ channel activation and inactivation rates,^39^^,^^40^ but also reduce long-closed states.^40^ Our work has shown that proteolytic cleavage of the pro-form of α_2_δ into mature α_2_δ acts as a permissive molecular switch for the function of Ca_V_1 and 2 channels.^41^ It should also be noted that although α_2_δ proteins increase the trafficking of Ca_V_ channels, they may also be able to traffic to the plasma membrane and to presynaptic terminals alone^41^ in the absence of calcium channels,^42^ and can have additional roles on synapse morphology.^43–45^

## Some Distinct Membrane Properties of Presynaptic Terminals

Presynaptic terminals generally have lower membrane excitability than axons, since voltage-gated Na^+^ channels are often more sparse than at nodes of Ranvier.^46^ In the presynaptic calyx of Held, Na^+^ channels are absent from the calyx terminal region, but concentrated in the final unmyelinated segment of axon (heminode) leading up to the calyx.^47^ The concentration of specific voltage-gated K^+^ channels, particularly inactivating K^+^ channels, controls presynaptic excitability,^46–49^ such that presynaptic action potentials are generally either brief,^47^ or attenuated.^46^ Other channels that may be present presynaptically, such as hyperpolarization-activated HCN channels, also have the ability to affect resting membrane potential.^50^ Although a recent study has highlighted that rapid Ca^2+^ entry can occur through tetrodotoxin-sensitive Na^+^ channels, which are highly concentrated in the axon initial segment,^51^ the sparsity of presynaptic Na^+^ channels means it is unlikely that this route contributes significantly to presynaptic Ca^2+^ entry.

The presynaptic membrane potential has been directly measured in several types of accessible terminals. For example, in the calyx of Held excitatory terminal, it was about −80 mV, and in the same study the resting intracellular Ca^2+^ was estimated to be about 50 nM.^52^ In hippocampal mossy fiber boutons, the resting membrane potential was between −60 and −85 mV,^48^ and in inhibitory Purkinje cell terminals in culture, the membrane potential was −69 mV.^46^ At these potentials even Ca_V_3 channels, if present, would show little tonic activity (Figure 1A).

## Implications of Different Presynaptic Ca_V_ Channel Compositions for Neurotransmitter Release

From the foregoing discussion, it is clear that the membrane potential of most presynaptic terminals is sufficiently negative that the vast majority of Ca_V_2 channels are closed, rather than inactivated in the absence of ongoing activity. Thus, Ca_V_2 channels are available to open upon action potential arrival. Ca_V_2.1 channels generally activate at similar potentials to Ca_V_2.2 in cell lines (Table 1), but activate more rapidly.^13^ However in calyx of Held synapses, presynaptic N-type I_Ca_ was found to activate ∼ 8 mV more depolarized than P/Q type current,^53^ and this was also seen in chromaffin cells.^54^ The third subtype of Ca_V_2 channel (Ca_V_2.3) also known as R-type has a somewhat more hyperpolarized membrane potential^14^ (Table 1), potentially pointing to differences in function.

For most synapses, Ca_V_2.1 (P/Q)- and Ca_V_2.2 (N)-type channels are involved in varying proportions in synaptic transmission, depending on the synapse in question and the developmental stage. Broadly, Ca_V_2.1 channels become of increasing importance in many synapses as they develop, such that they predominate in some mature neurons,^53^^,^^55^ and are also more tightly associated with the release machinery^55^ (see below). At some synapses, Ca_V_2.3 channels, activated by smaller depolarizations, play an important role, rarely as the main channel involved in vesicular release, although this is the case in habenula cholinergic neuron terminals in the interpeduncular nucleus.^56^ More often Ca_V_2.3 has been found to underlie other processes such as delayed or asynchronous release, for example from small hippocampal boutons,^57^ and it also plays a role in long-term potentiation.^58^

A key factor to consider is action potential duration, relative to the rate of deactivation of the calcium channels, as much of the Ca^2+^ entry mediating synchronous release will occur on the repolarization phase of each brief action potential-mediated presynaptic depolarization, which has the effect of increasing the driving force for Ca^2+^. In contrast, asynchronous release is the term for release resulting from stochastic opening of individual channels near the membrane potential, often after a burst of action potentials,^57^^,^^59^ resulting in long-duration presynaptic Ca^2+^ transients. Although it has been suggested that spontaneous openings of Ca_V_2.3 channels may be in part responsible for asynchronous release occurring after action potentials at some synapses,^57^ Ca_V_2.1 and Ca_V_2.2 channels, particularly when associated with the ß2a subunit which reduces their inactivation, may also play a role.^59^ For example, at synapses formed by different subtypes of hippocampal GABA-ergic interneuron, Ca_V_2.1 is involved in the mainly synchronous release from fast-spiking parvalbumin interneurons, whereas Ca_V_2.2 channels predominantly mediate GABA release from cholecystokinin-containing interneurons, of which a much greater fraction is asynchronous release.^60^

At some specialized sensory synapses, L-type channels, particularly Ca_V_1.3 and Ca_V_1.4, are critical for function. These mainly concern the auditory inner hair cells (Ca_V_1.3)^61^^,^^62^ and retinal photoreceptors and bipolar neurons (Ca_V_1.4),^11^^,^^63^^,^^64^ in which the presynaptic responses are graded. These particular Ca_V_1 channels have properties suited to this function, in that they remain available at depolarized potentials (Figure 1C).

## Concerted Calcium Channel Involvement in Release from Individual Synapses

As described above, both Ca_V_2.1 and Ca_V_2.2 calcium channels are involved, to varying extents, in vesicular release at most individual central nervous system terminals, as judged by ω-agatoxin IVA and ω-conotoxin GVIA inhibition, respectively.^65–67^ However, the relative amount of block by each toxin cannot be used directly to determine the prevalence of these channels, because of the nonlinear, approximately fourth power, relationship between intracellular Ca^2+^ levels and neurotransmitter release.^68–72^ There are several related forms of Ca^2+^ cooperativity that have been described, that between multiple Ca_V_ channels required to release a single vesicle^73^ and the number of Ca^2+^ ions that must bind cooperatively to Ca^2+^ sensors, and the cooperative action of those sensors, to trigger release of a vesicle.^74^

Thus, there is generally found to be synergy between the opening of multiple channels to reach the µM levels of Ca^2+^ at the Ca^2+^ sensors whose occupancy mediates release of each vesicle in an active zone. The numbers of channels involved have been estimated to be very small in some synapses^67^^,^^75–77^, to over 60 in immature calyx of Held synapses.^78^ In a few cases a single channel has been found to be sufficient,^75^^,^^76^ although the probability of release will be low.^77^ The number of channels present in each active zone is much greater than those that open in response to each action potential, because of the low probability of opening of each channel and the stochastic nature of channel openings, meaning they occur with a variable delay following a depolarizing stimulus, which can also lead to failure of exocytosis.

## Anchoring of Calcium Channels in Presynaptic Active Zones is Key to Their Differing Roles in Synaptic Transmission

**Figure 2.**
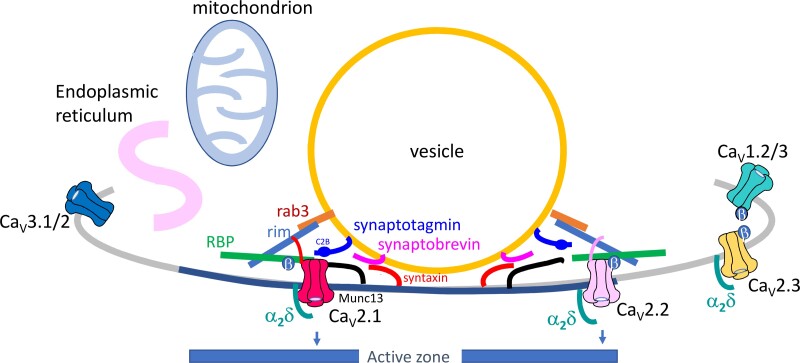
Diagram of Ca_V_ Channels in Relation to Other Pre-Synaptic Proteins and Organelles. Some of the proteins involved in anchoring Ca_V_ channels near to synaptic vesicles forming a nanodomain within the presynaptic active zone (dark blue membrane). These include Rab3 (orange), synaptotagmin (purple), and synaptobrevin (pink) associated with the vesicular membrane. Rim (blue) and RBP (green) are cytosolic; Munc13 (black) and syntaxin (orange) are associated with the plasma membrane. Ca_V_2.1 (red) and Ca_V_2.2 (light pink) are likely to be differentially localized within active zones, whereas the other Ca_V_ channels, if present, are thought to be located elsewhere in the presynaptic membrane. Figure based on Fig. 4a in Dolphin and Lee.^81^

The proximity of the presynaptic Ca_V_ channels to the vesicular release site is an extremely important factor in determining the properties and speed of neurotransmitter release. In order to study this, knowledge of the relative locations of the channel subtypes, as well as modeling studies are required, in addition to an understanding of the biophysical and biochemical distinctions between Ca_V_2.1 and Ca_V_2.2 channels.^77^^,^^79^ There are well-studied differences in the anchoring of the two main Ca_V_2 channels in presynaptic active zones. Both Ca_V_2.1 and Ca_V_2.2 channels are tethered in active zones by the RAB3A-interacting molecule (RIM),^80^ and Ca_V_2.3 channels may also associate with RIM proteins.^80^ Furthermore, RIM-binding protein interacts with Ca_V_2.1, Ca_V_2.2 and Ca_V_1.2 channels, but recruits only the former two channels via interaction with RIM specifically to the active zone.^80^ However, Ca_V_2.1 is selectively associated with certain Munc13 isoforms potentially leading it to be localized closer to docked vesicles than Ca_V_2.2^55^ (Figure 2). In contrast to the obvious central phenotype of Ca_V_2.1 knockout mice,^82^ the lack of marked phenotype in Ca_V_2.2 knockout mice suggests that their role is less crucial, and other types of Ca_V_ channel (particularly Ca_V_2.1) are able to compensate for the loss of Ca_V_2.2 at most synapses. However, Ca_V_2.2 channels have a predominant role at primary afferent synapses in the pain pathway,^83^^,^^84^ and this pathway is indeed disrupted in Ca_V_2.2 knockout mice.^85^

Thus, both the properties and distribution of Ca_V_2.1 channels result in greater activation and Ca^2+^ entry for a brief action potential through these channels than for Ca_V_2.2.^13^ This has been observed, for example, in mossy fiber boutons, where a single terminal was estimated to contain about 2000 channels, and brief presynaptic action potentials activated a presynaptic Ca^2+^ current that was found with pharmacological blockers to be dependent on P/Q (∼66%), N (∼26%), and R (∼8%)-type channels.^86^

Other proteins have also been found to interact with Ca_V_2 channels,^87^ and some of these proteins affect the properties of the channels, such as the CRMP-2 interaction with Ca_V_2.2.^88^ Another presynaptic protein, Syntaxin 1A has been found to interact with part of the II-III linker of Ca_V_2.2 channels (synprint site), increasing both slow inactivation and steady-state inactivation, and thus reducing channel availability.^89^^,^^90^ By contrast, an analogous effect on Ca_V_2.1 channels may depend on channel splice variant.^91^ In presynaptic terminals, this could affect the relative availability of Ca_V_2.1 and Ca_V_2.2 channels. However, this synprint site is not essential for presynaptic targeting^92^ or neurotransmission.^93^

## Ca_V_2 Channel Modulation Dramatically Affects Their Presynaptic Function

Since Ca_V_2 channels are subject to inhibition by several second messenger pathways, this will affect their availability. Thus, the integral of Ca^2+^ entry at any synapse depends on a multitude of factors that are unique to each condition and to the pattern of action potentials arriving at the terminal. In particular, G-protein-mediated inhibition is an important property of Ca_V_2 channels. This can result from stimulation of many presynaptic G-protein coupled receptors linked to G_i/o_, such as GABA-B receptors, opioid receptors, and others whose activation leads to the release of Gßγ subunits.^94–99^ This inhibition, which may have a tonic component, shifts the voltage-dependence of Ca_V_2 channel activation to more positive potentials, and slows activation kinetics,^100^^,^^101^ which can be overcome by prior depolarization, including in some cases an action potential train.^102^ This macroscopic current slowing is mediated at the single-channel level by a prolongation of the latency to first opening both of native N-type single-channel currents^103^ and of cloned Ca_V_2.2 channels,^104^^,^^105^ with no change in single-channel conductance.

Gßγ binding mediates the inhibition, and voltage-dependent Gßγ unbinding underlies the slow activation of the Ca_V_2 channels, and triggers the depolarization-mediated reversal of inhibition.^96^^,^^98^ Here it should be noted that Ca_V_2.1 channels are less subject to G-protein modulation than Ca_V_2.2, since the Gßγ off-rate from these channels is more rapid.^98^

Given that, as described above, only a few Ca_V_ channels may open in response to a single action potential at individual synapses, and Gßγ-mediated inhibition involves slowing of their activation, the effect on synaptic transmission has the potential to be profound, particularly where a high proportion of Ca_V_2.2 channels is present, such as primary afferent terminals.^6^

## T-type Channels Are Partially Inactivated at Resting Membrane Potentials

T-type channels are present in certain presynaptic terminals, and they may play an important role in influencing resting Ca^2+^ levels, or in providing Ca^2+^ for downstream events. Although Ca_V_3 channels do not normally supply significant amounts of Ca^2+^ for neurotransmitter release resulting from action potentials arriving at the terminal, nevertheless their availability can be affected by the interplay of other channels such as HCN channels and Ca^2+^-activated K^+^ channels, which affect membrane potential.^50^ Functional HCN1 channels are present on particular glutamatergic synaptic terminals, for example onto entorhinal cortical layer III pyramidal neurons, where they depolarize the membrane potential and reduce neurotransmitter release. These effects at least partly result from reduced availability of Ca_V_3.2 channels.^50^ Furthermore, Ca_V_3 channels were also found to play an important part in asynchronous dendrodendritic release of glutamate from olfactory bulb mitral cells.^107^ In another study GABA release from interneurons could be promoted by activation of presynaptic nicotinic receptors and subsequent activation of presynaptic Ca_V_3.1 channels, together with release of Ca^2+^ from ryanodine-sensitive intracellular stores.^108^ Thus, there is evidence from numerous studies for a variety of presynaptic roles for T-type channels.

## A Role for Ca^2+^-induced Ca^2+^ Release in Presynaptic Terminals

Although Ca^2+^-induced Ca^2+^ release (CICR) is mainly associated with Ca_V_1.2 channel function, for example in cardiac muscle cells, nevertheless smooth endoplasmic reticulum is present in presynaptic terminals,^109^ and there is evidence that CICR occurs from this endoplasmic reticulum which can affect neurotransmitter release.^110^^,^^111^ The channels involved in presynaptic CICR are mainly ryanodine receptors,^112^^,^^113^ and the initial source of Ca^2+^ for CICR could be Ca_V_ channels, particularly T-type or R-type, which are activated by small depolarizations,^113^ or other presynaptic Ca^2+^-permeable channels such as α7 nicotinic receptors.^108^^,^^114^ It was further suggested that clustering of the endoplasmic reticulum sensor of Ca^2+^ depletion, STIM1, may directly inhibit Ca_V_ channels.^110^ The importance of CICR in neurotransmitter release is more evident following prolonged activation rather than single action potential-induced responses,^111^^,^^113^ although single action potentials can also result in CICR.^111^^,^^115^

## The Roles of Mitochondria in Controlling Intracellular Ca^2+^ in Presynaptic Terminals

Mitochondria are present in about half of all presynaptic terminals,^116^ and they can sequester presynaptic Ca^2+^ entry resulting from trains of action potentials.^117^^,^^118^ Presynaptic mitochondria are found to have a low threshold for Ca^2+^ uptake, relative to those in other tissues, which is conferred by a brain-specific protein MICU3, allowing mitochondria to take up Ca^2+^ directly from the cytoplasm near to sites of Ca^2+^ entry through the plasma membrane.^119^ Indeed, mitochondria have been visualized to be tethered to presynaptic terminal membranes in the calyx of Held.^120^ Furthermore, Ca^2+^ is required for optimal ATP levels, and presynaptic mitochondria promote synaptic transmission in active synapses by supplying the essential ATP. Maintenance of the voltage and ionic gradients related to presynaptic function is also a major consumer of ATP,^119^ and thus mitochondria fulfill multiple presynaptic roles.

## Conclusions

The molecular and biophysical properties of Ca_V_ channels are finely tuned to their roles in presynaptic terminals to mediate neurotransmitter release. Although there are many types and geometries of synapse, the channels in these terminals function in broadly similar ways to mediate Ca^2+^ entry that triggers vesicular release. Since the opening of a few channels, or even a single channel, is able to mediate release at discrete small excitatory and inhibitory synapses, it is extremely important to understand the individual and distinct properties of these channels, in order to appreciate how this process of release is constrained by the localization, tethering, properties, and modulation of the channels. Similarly, the different mix of types of channels present, and their relative active zone distribution, is tuned to the functions of individual synapses and to changes during development and synaptic activity.
